# Interactions mediated by a public good transiently increase cooperativity in growing *Pseudomonas putida* metapopulations

**DOI:** 10.1038/s41598-018-22306-9

**Published:** 2018-03-06

**Authors:** Felix Becker, Karl Wienand, Matthias Lechner, Erwin Frey, Heinrich Jung

**Affiliations:** 1 Microbiology, Department Biology 1, Ludwig-Maximilians-Universität Munich, Grosshaderner Strasse 2-4, D-82152 Martinsried, Germany; 2 Arnold-Sommerfeld-Center for Theoretical Physics and Center for Nanoscience, Ludwig-Maximilians-Universität, Theresienstrasse 37, D-80333 Munich, Germany

## Abstract

Bacterial communities have rich social lives. A well-established interaction involves the exchange of a public good in *Pseudomonas* populations, where the iron-scavenging compound pyoverdine, synthesized by some cells, is shared with the rest. Pyoverdine thus mediates interactions between producers and non-producers and can constitute a public good. This interaction is often used to test game theoretical predictions on the “social dilemma” of producers. Such an approach, however, underestimates the impact of specific properties of the public good, for example consequences of its accumulation in the environment. Here, we experimentally quantify costs and benefits of pyoverdine production in a specific environment, and build a model of population dynamics that explicitly accounts for the changing significance of accumulating pyoverdine as chemical mediator of social interactions. The model predicts that, in an ensemble of growing populations (metapopulation) with different initial producer fractions (and consequently pyoverdine contents), the global producer fraction initially increases. Because the benefit of pyoverdine declines at saturating concentrations, the increase need only be transient. Confirmed by experiments on metapopulations, our results show how a changing benefit of a public good can shape social interactions in a bacterial population.

## Introduction

Bacteria have complex social lives: they communicate with each other and with other organisms, form tight communities in biofilms, exhibit division of labor, compete, and cooperate^1–7^. They also produce and exchange public goods. Public goods are chemical substances that are synthesized by some individuals (known as *producers* or *cooperators*) and are then shared evenly among the whole population, including cells that did not contribute to their production^8–10^. Such social interactions can also influence population dynamics, as exemplified in the context of metapopulations^11–17^. Metapopulations consist of several subpopulations. The subpopulations may grow independently for a time, then merge into a single pool that later splits again, restarting the cycle. This ecological system, which mimics some bacterial life-cycles^18,19^, also dramatically impacts the population’s internal dynamics. To mathematically analyze the effects of social interactions, they can be framed in terms of game theoretical models^20–24^ –for instance, the prisoner’s dilemma, in the case of the exchange of public goods^25–28^ –or formulated in terms of inclusive fitness models^29–31^. These approaches underestimate the impact on the social interaction of specific properties and mechanisms of action of the public good in question, mostly to simplify the mathematical description. Previous investigations have shown that, for example, phenomena like public good diffusion^32–34^, interference of different public goods with each other^35^, the regulatory nature of public good production^36^, or its function in inter-species competition^37^ may affect strain competition. The shortcomings of game-theoretical models in studying the evolution of cooperation can be overcome by systems biology modeling approaches^34,38,39^.

In this work, we directly quantify a social interaction mediated by a public good. Thus, we adopt a systems biology approach, rather than a more reductive game-theoretical one. We focus on the dissemination of iron-scavenging pyoverdine (PVD) in a metapopulation of fluorescent *Pseudomonas putida*, and study how its biological function determines the population dynamics.

**Figure 1:**
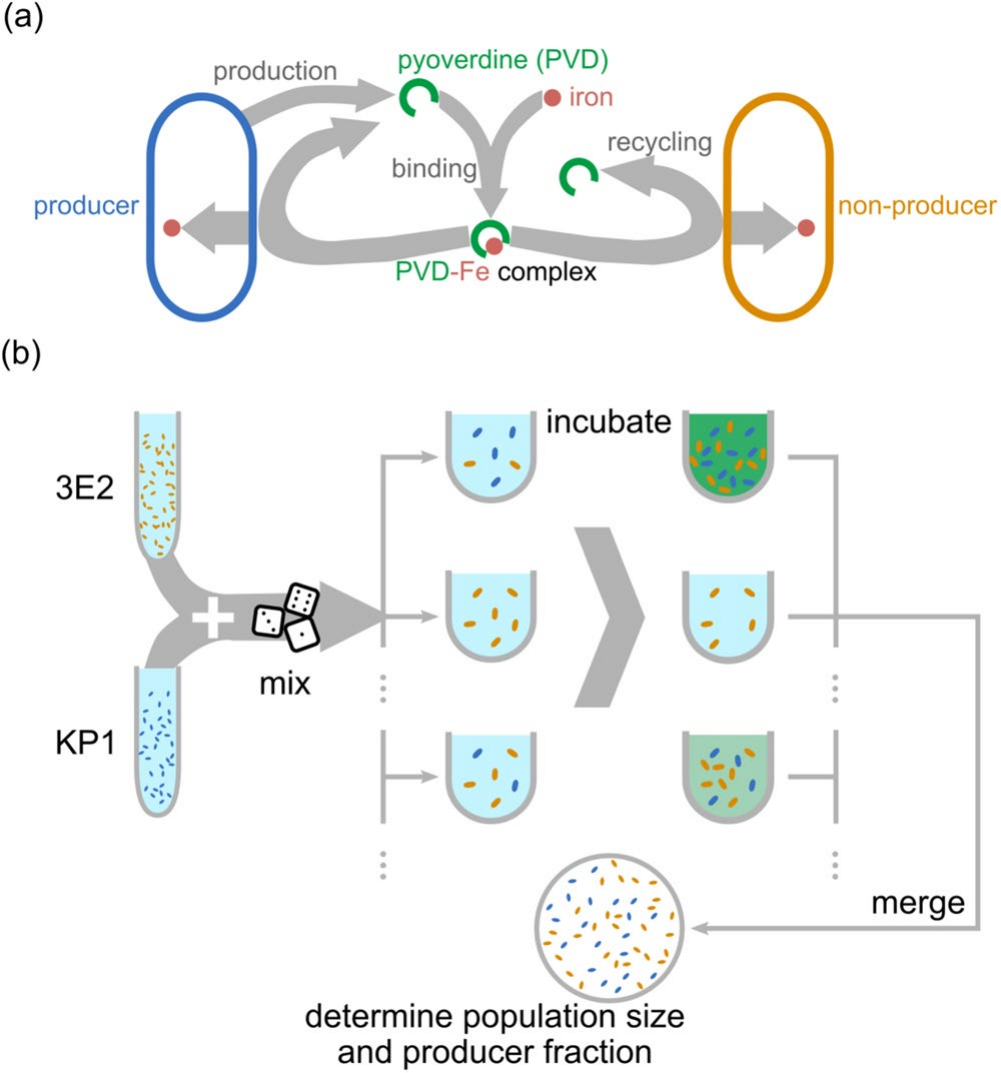
Outline of PVD-mediated interactions and experimental setting. (**a**) Outline of the social interaction. Producers (blue) secrete pyoverdine (PVD, green) into the environment, where it binds iron (red). The resulting Fe-PVD complex is transported into the periplasm of both producers and non-producers. Iron is reduced and incorporated into cells, while PVD is transported back into the environment to scavenge additional ferric ions^44–46^. (**b**) Metapopulation growth setting. We initiate a metapopulation by mixing producers and non-producers in random proportions and inoculating the individual populations, which grow independently. At given time points ${t}$, we take samples from each population, and merge them to determine the average population size and the global producer fraction of the metapopulation.

In this well-established, native model system, cells secrete PVD into the environment to facilitate iron uptake when the metal becomes scarce^29,40–44^. PVD binds to ferric iron and is then actively transported into the periplasm. There, the iron is reduced, released and transported across the plasma membrane, while PVD is secreted back into the environment^44–46^. Figure 1 outlines the PVD-mediated interaction between producer and non-producer cells and the metapopulation set-up we use to study its effects on population dynamics.

In the following, we show, both experimentally and in computer simulations, that the global fraction of producer cells across a metapopulation increases during growth, but only transiently. This effect hinges on the specifics of PVD biochemistry, which elude a game-theoretical analysis. Thus, our study shows that the specific features of the public good considered are the key determinant of the outcome of the social interaction. Our experiments employ a well-defined system, with a constitutive producer and a non-producer strain. The simulations use a mathematical model based on quantitative measurements of PVD’s costs and benefits, as well as its behavior as an accumulating public good. For appropriate values of the parameters, the theoretical results match those of experiments with *P. putida* metapopulations.

## Results

### Characterization of the model system

To investigate the social role of public goods, we chose the soil bacterium *P. putida* KT2440 as a model system. This is a well-defined system in which, as sketched in Fig. 1a, a single public good mediates all cell-cell interactions. *P. putida* KT2440 synthesizes a single type of siderophore – a pyoverdine (PVD) molecule^47^ –and does not produce 2-heptyl-3-hydroxy-4-quinolone or other known quorum-sensing molecules that might otherwise interfere with the social interaction^48–50^.

**Figure 2:**
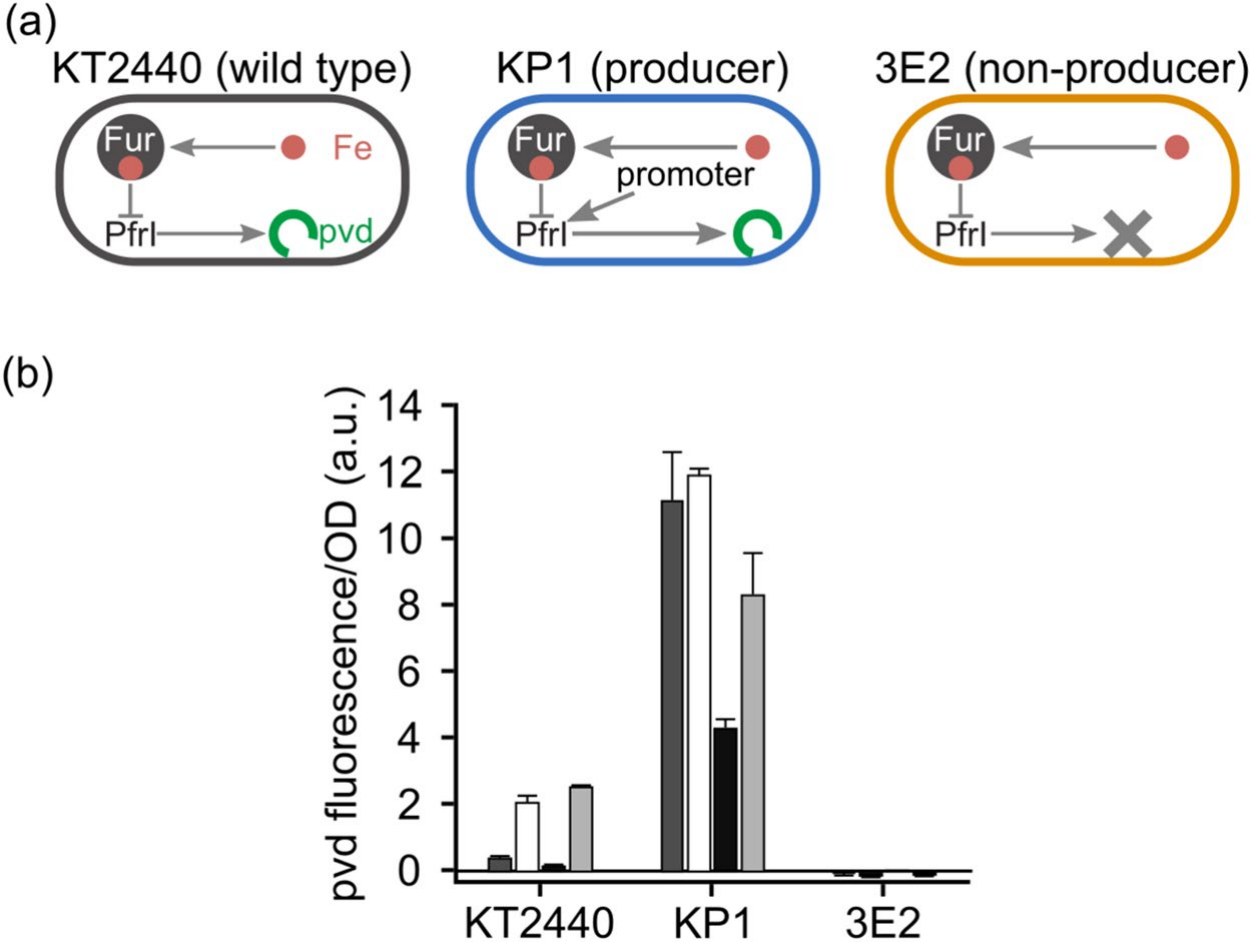
Characterization of the strains. (**a**) Sketch of each strain’s regulatory system. In the wild-type *P. putida* KT2440 (gray), the ferric uptake regulator Fur binds iron and represses the expression of the *pfrI* gene necessary for PVD synthesis. The constitutive producer strain KP1 (blue) carries an additional copy of the *pfrI* gene controlled by a constitutive promoter. The non-producer strain 3E2 (orange) has an inactivated non-ribosomal peptide synthetase gene, which prevents PVD synthesis. (**b**) Average PVD production per cell by the wild-type and strains KP1 and 3E2 after 8 h of cultivation. The darker the columns, the more abundant is the iron in the medium. Dark gray columns represent moderate iron availability conditions (KB medium without additions); white columns represent extreme iron limitation (KB/1 mM DP); black columns represent iron-replete conditions (KB/100 µM FeCl_3_); light gray columns represent iron-limiting conditions (KB/100 µM FeCl_3_/1 mM DP). KP1 produces PVD under all conditions (albeit with different yields), 3E2 never produces the siderophore, and the wild-type adapts its rate of synthesis to iron availability.

Wild-type *P. putida* KT2440 controls PVD production through a complex regulatory network. As shown in Fig. 2a, the central element of the network is the ferric uptake regulator (Fur) protein, which binds iron and, among other things, down-regulates expression of the iron starvation sigma factor *pfrI*^51–53^, which in turn directs the transcription of PVD synthesis genes. As a consequence, siderophore production continually adapts to the availability of iron^47,52^. This regulation, however, obscures the costs of PVD production, as it also affects other processes. We therefore circumvented it by generating a *P. putida* strain, called KP1, which constitutively produces PVD. KP1 carries a copy of the *pfrI* gene controlled by the constitutive promoter P_*A1/04/*03_^54^ at the *att*Tn7 site in the KT2440 genome. As the non-producer, we used strain 3E2, which carries an inactivated non-ribosomal peptide synthetase gene (pp4220) that inhibits PVD synthesis^47^. The two strains were otherwise isogenic.

We characterized producer (KP1) and non-producer (3E2) strains by measuring their average per-cell PVD production under different iron availabilities, and comparing the results with those for the wild type (strain KT2440). We cultivated all three strains, separately, in KB medium and KB supplemented with 100 µM FeCl_3_ (for short, KB/100 μM FeCl_3_), as well as in the same two media supplemented with the chelator dipyridyl (DP, 1 mM) to reduce iron availability. Using atom absorption spectroscopy, we determined an iron concentration in KB of about 8 µM. Figure 2b shows the average amount of PVD produced per cell after 8 h of growth (close to the end of exponential growth). The wild type partially represses production of PVD under moderate iron availability (KB, dark gray bars), and ceases synthesis altogether under high iron availability (KB/100 μM FeCl_3_, black bars). Addition of DP reduces iron availability and stimulates PVD production in both media (Fig. 2b, white bars: KB/1 mM DP, light gray bars: KB). In contrast, KP1 produces large amounts of PVD under all tested growth conditions, and thus represents a constitutive PVD producer. The yield depends on conditions, probably because the regulated copy of *pfrI* is still present in the genome. 3E2, finally, never synthesizes PVD, regardless of the conditions, and is a true non-producer, as previously reported^47^.

### Quantifying the social role of pyoverdine

Having established how each strain behaves, we quantified the impact of PVD on population dynamics. Specifically, we wanted to determine the metabolic load of PVD production, its impact on growth, its stability, and how evenly it is shared with other cells.

**Figure 3:**
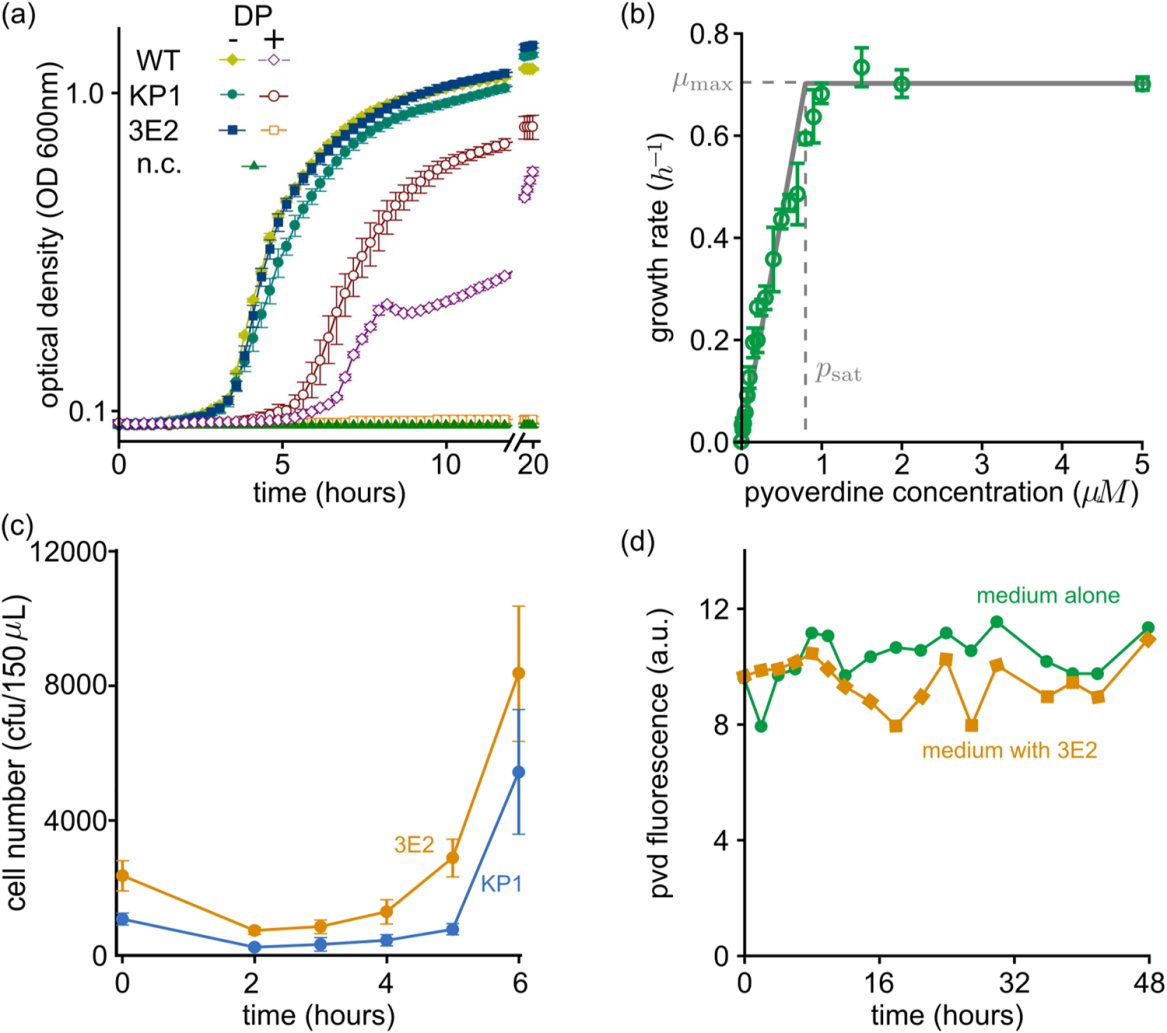
Characterization of the social impact of pyoverdine (PVD) in terms of costs (**a**), benefits (**b**), degree of sharing among cells (**c**) and stability (**d**). (**a**) In an environment with available iron (KB, solid symbols), non-producer cells (strain 3E2) grows as fast as the wild-type (WT), and faster than the producer (strain KP1). Under extreme iron limitation (KB /1 mM DP, empty symbols), PVD is needed for growth: KP1 and WT grow, whereas 3E2 does not (mean values and standard deviations were calculated from six measurements). (**b**) Green dots represent the growth rate $\mu$ of 3E2 cultures, measured under extreme iron limitation (KB/1 mM DP) in the presence of the indicated concentrations of added PVD (error bars are standard deviation over four replicates). The solid gray line represents the growth rate calculated using equation (2) (maximal growth rate ${\mu }_{{\rm{\max }}}$ and the saturation concentration ${p}_{{\rm{sat}}}$ fitted to the experimental data: ${\mu }_{{\rm{\max }}}=0.878$, ${p}_{{\rm{sat}}}$ = 0.8). (**c**) Early growth of KP1–3E2 co-cultures (initial fraction of KP1 = 0.33) under extreme iron limitation (KB/1 mM DP). Shown are mean and SD of eight independent experiments. Since 3E2 needs PVD to grow (see panel **b**), this result indicates that PVD is shared between the strains. (**d**) Stability of PVD (2 µM) in KB medium and in the presence of the non-producer 3E2. The fluorescence emission was recorded at 460 nm (excitation 400 nm).

We assessed the impact of PVD production on growth by comparing the growth rates of strains KP1 and 3E2 under iron-rich conditions (KB). As shown in Fig. 2b, neither 3E2 nor the wild-type produces substantial amounts of PVD under these conditions, and the solid symbols in Fig. 3a show that both strains grow at about the same rate. KP1, on the other hand, produces PVD and grows more slowly. The data in Supplementary Table S1 allow us to quantify this difference in growth rate. Depending on the conditions, KP1’s growth rate is 3–10% lower than that of strain 3E2. For example, the difference is minimized (1.03-fold) when the medium is replaced (in a 24-well plate format) every hour, whereas the largest difference (1.10-fold) is observed in batch cultures (96-well plate format). This suggests that factors other than iron level *per se*, such as nutrients and oxygen availability, modify the metabolic impact of PVD production.

The empty symbols in Fig. 3a illustrate the growth of the strains under extreme iron limitation (KB/1 mM DP). In these conditions, PVD is indispensable for iron uptake, and only producing strains – KP1 and the wild-type – can grow at all. Less restrictive conditions (KB/100 μM FeCl_3_ and KB/100 μM FeCl_3_/1 mM DP) produce qualitatively similar results (see Supplementary Fig. S1). 3E2, if cultivated alone, cannot grow unless the medium is supplemented with PVD isolated from a producer culture. Figure 3b shows the maximal growth rate of 3E2 under these conditions as a function of the concentration of added PVD. For values lower than about 1 μM, the growth rate increases almost linearly with PVD concentration, then sharply levels off. Higher PVD concentrations do not further stimulate growth – which is consistent with observations of iron saturation in other bacterial systems^55,56^.

This saturating behavior, we argue, stems directly from PVD’s ability to bind iron and make it available to cells. Because PVD has an extremely high affinity for iron [10^24^ M^−1^ for Fe^3+^ at pH 7^57^, we can assume that each PVD molecule immediately binds an iron ion. Therefore, the PVD concentration $p$ is equivalent to that of PVD-Fe complexes, and represents the concentration of iron accessible to cells (this may not hold if the level of PVD exceeds that of the iron available, but we expect this extreme case to arise only after the exponential growth phase in our setting, if ever). Each cell, then, incorporates iron ions at a constant rate $k$ ·$p$ which is proportional to the PVD concentration $p$. Moreover, cells try to maintain a constant internal iron concentration $F{e}_{{\rm{in}}}$ and reproduce at a PVD-dependent rate $\mu (p)$ when growth is limited by iron availability. If we also assume that the cell volume just before division is twice the volume $V(0)$ of a newborn cell, we find that the growth rate is proportional to $p$ (see Supplementary Note):

1 $$\mu (p)=\frac{k}{F{e}_{{\rm{in}}}V(0)}p.$$

For PVD concentrations above 1 μM, however, some other factor limits growth. Cells cannot further increase $\mu (p)$, regardless of the PVD concentration, and the benefit of PVD saturates. In summary, there is a limit PVD concentration ${p}_{{\rm{sat}}}$ (~1 μM), below which the growth rate is proportional to the PVD concentration, following equation (1). Above ${p}_{{\rm{sat}}}$, the growth rate is a constant ${\mu }_{{\rm{\max }}}$, whose value depends on the culture conditions. In mathematical terms,

2 $$\mu (p)={\mu }_{{\rm{\max }}}min(\frac{p}{{p}_{{\rm{sat}}}},1)=\{\begin{array}{c}\frac{{\mu }_{{\rm{\max }}}}{{p}_{{\rm{sat}}}}p,\,if\,p < {p}_{{\rm{sat}}}\\ {\mu }_{{\rm{\max }}},\,if\,p\ge {p}_{{\rm{sat}}.}\end{array}$$

The gray curve in Fig. 3b shows the function described in equation (2). Fitting the values for the parameters ${p}_{{\rm{sat}}}$ and ${\mu }_{{\rm{\max }}}$, the curve closely resembles the experimental results, validating our argument.

A central question in determining the social role of PVD is whether cells share the molecule with other cells, and thus also its benefit, or keep it to themselves. In other words, to what extent is PVD a *public* good? Fig. 3c shows the early stages of growth of a mixed population of KP1 and 3E2 (initial fraction of KP1 = 0.33) under extreme iron limitation (KB/1 mM DP). After a lag phase of about 2 h, both strains begin to grow. Since 3E2 needs PVD to grow in these conditions (see above and Supplementary Fig. S1), we conclude that both strains receive the benefit at the same time, and neither has preferential access to it. In our experiments, then, PVD behaves as a truly public good. Consequently, populations that start with a higher producer fraction ${x}_{{\rm{0}}}$ have more PVD available, and grow faster than populations with low ${x}_{{\rm{0}}}$ values (as shown in Supplementary Figs S1 and S2).

PVD is also very stable. Figure 3d shows the fluorescence yield of PVD over 48 h in KB medium alone (green line). The value fluctuates around a constant average, indicating that PVD does not spontaneously degrade – at least not appreciably – within the time scales of our experiments. The orange line in Fig. 3d represents a similar measurement, but in the presence of non-producer cells. In this case also, fluorescence does not appreciably decay, so cells do not seem to consume PVD during the interaction. This also means that, provided producers are present, the public good accumulates in the environment once its synthesis has been triggered.

Taken together, these observations characterize the social interaction as follows: (i) Constitutive producers grow more slowly than non-producers (given equal PVD availability); (ii) PVD acts as a public good, which is homogeneously shared among cells; (iii) once produced, PVD persists: it is chemically and functionally stable, and cells recycle it rather than consuming it; (iv) the public good drives the population dynamics, since PVD is necessary for access to the iron required for growth.

### Modeling social and growth dynamics

Based on the experimental results presented in the previous section, we formulated a set of equations to describe the development of a single, well-mixed population of $c$ producers and $f$ non-producers. The population dynamics follows a logistic growth, where the function $\mu (p)$ from equation (2) determines the per-capita growth rate. For our experimental setup, we estimate cells to incorporate only a minimal fraction of the available iron (<3%, see Supplementary Note), so the assumptions of equation (2) hold (and some other resource determines the carrying capacity $K$. Although KP1 synthesizes PVD at condition-dependent rates, we adopt a simplified description and model synthesis as occurring at a constant rate $\sigma$. The produced PVD does not decay but accumulates in the medium. Finally, the costs of PVD synthesis slow down the growth of producers by a factor ${\rm{1}}-s$ (where ${\rm{s}} < 1$), compared to non-producers. All in all, assuming the interaction between cells and PVD is fast, the dynamics can be summarized in the following equations:

3 $$\begin{array}{ccl}\frac{dc}{dt} & = & c\mu (p)(1-s)(1-\frac{c+f}{K}),\\ \frac{df}{dt} & = & f\mu (p)(1-\frac{c+f}{K}),\\ \frac{dp}{dt} & = & \sigma c.\end{array}$$

This set of equations mathematically describes the experimental facts, in terms of measurable quantities. It is also different from a traditional game theoretical formulation, which would require us to somehow define a payoff function.

To better highlight the key factors of the population dynamics, we rescale the variables in equations (3). First, we measure population size in terms of the fraction of resources used up, i.e., $n:=(c+f)/K$. This definition means that $K$ determines the scale of population sizes, while $n$ takes values between 0 and 1: as *n* approaches 1, the resources become depleted, and cells enter a dormant state^14^. Second, we consider the fraction $x:=c/(c+f)$ of producers within each population, rather than their absolute number. Third, we measure the amount of PVD in units of the saturation concentration, $v:=p/{p}_{{\rm{sat}}}$ (and define $\mu (v)=min(v,1)$). Finally, measuring time in units of the minimal doubling time $1/{\mu }_{{\rm{\max }}}$, equations (3) become

4 $$\begin{array}{ccl}\frac{dn}{dt} & = & n(1-n)(1-sx)\mu (v),\\ \frac{dx}{dt} & = & -sx(1-x)(1-n)\mu (v),\\ \frac{dv}{dt} & = & \alpha nx,\end{array}$$

where $\alpha :=\frac{\sigma K}{{p}_{{\rm{sat}}}{\mu }_{{\rm{\max }}}}$ is a dimensionless parameter. This parameter represents the rate at which PVD benefit saturation sets in. Keeping other factors constant, the benefit saturates sooner if production is faster (higher $\sigma$) and/or the number of total producers increases (higher $K$ and thus larger populations). Conversely, if the saturating PVD concentration is higher (higher ${p}_{{\rm{sat}}}$), or cell reproduction is faster (higher ${\mu }_{{\rm{\max }}}$), populations can reach higher densities before the benefit saturates. Generally speaking, the lower $\alpha$, the more advantageous producers are for their population. For $\alpha \to 0$ for example, the reproduction time scale is shorter than that of public good production. Therefore, the relatively scarce PVD strictly limits growth, PVD saturation occurs only after many generations, and producer-rich populations outgrow producer-poor communities for longer. At the other extreme, $\alpha \to \infty$ means that cells produce PVD much faster than they grow. In this case, a handful of producers suffices to quickly reach saturation levels of PVD. Whether they include few or many producers, all populations grow at the same rate, which negates the advantage of higher producer fractions.

**Figure 4:**
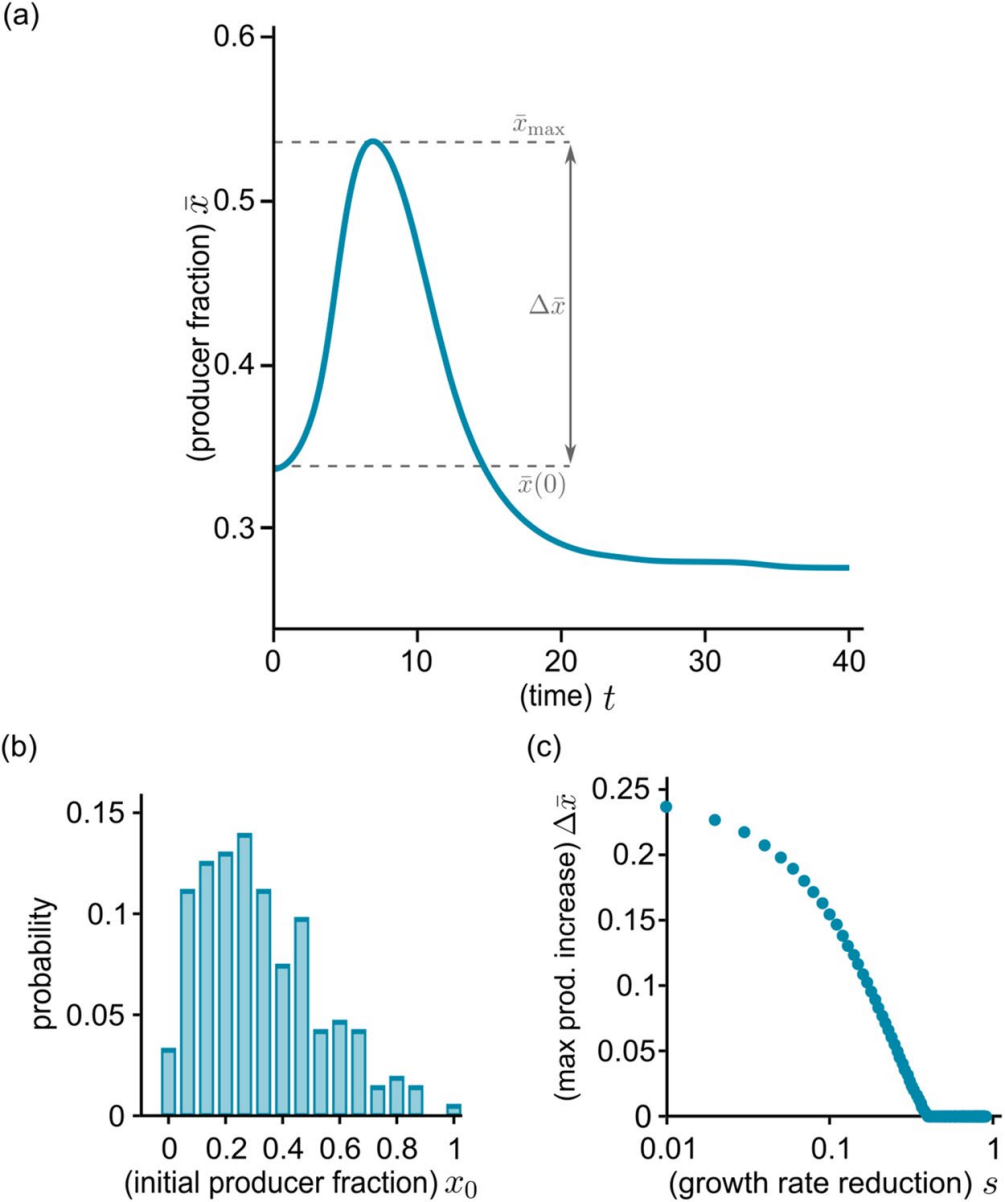
Simulation results for the growth of a metapopulation. The time course of the global producer fraction $\bar{x}$ (**a**) is computed by numerically solving equations (4) for a given distribution of stochastic initial compositions (**b**) (parameter values: $\alpha$ = 200, $s$ = 0.05, initial size ${n}_{0}={10}^{-3}$). The producer fraction initially increases as populations with more producers begin to expand earlier (see also Supplementary Movie S1). After reaching a maximum value ${\bar{x}}_{{\rm{\max }}}$, the global producer fraction decreases. (**c**) The maximal magnitude of the increase ${\rm{\Delta }}\bar{x}={\bar{x}}_{{\rm{\max }}}-\bar{x}(0)$ decreases with stronger growth reduction $s$ ($s$ between 0.01 and 0.9, other parameters identical to panel (**a**): for low $s$ it is comparable to the initial producer fraction, while very low producer growth precludes any increase at all.

**Figure 5:**
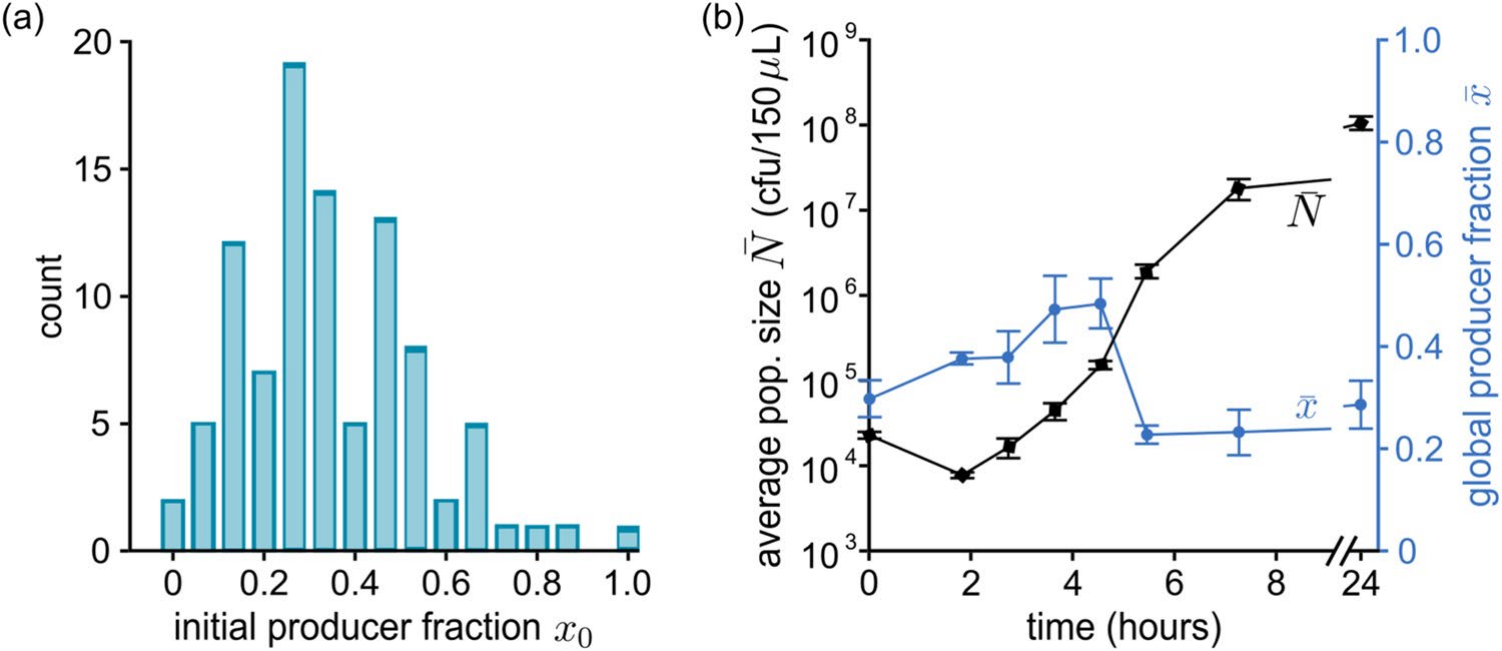
Experimental results for the growth of a mixed metapopulation. (**a**) Sample distribution of initial producer fractions in a 96-well plate. (**b**) Time course of the development of the total cell number $\bar{N}(t)$ and global producer fraction $\bar{x}(t)$ for a metapopulation grown under extreme iron limitation (KB/1 mM DP) in a 96-well plate shaken at 30 °C. At given time intervals, samples are taken from the wells, merged: $\bar{N}(t)\,\,$is determined by counting cfu and $\bar{x}(t)$ is assessed based on the (green) color of colonies. Error bars are the result of three to five determinations of the respective parameter at the given time point. After a lag phase, populations begin to grow exponentially. During this phase, the global producer fraction transiently increases, dips sharply, then stabilizes to its final value.

We can also use equations (4) to describe a metapopulation of $M$ independent populations. To simulate this scenario, we solve equations (4) numerically for an ensemble of stochastic initial conditions (using $M={10}^{4}$). We generate a stochastic distribution of initial producer fractions ${x}_{{\rm{0}}}$ – depicted in Fig. 4b – as implemented in the experiments (see Fig. 5a and Materials and Methods). Because the experiments described here deal with relatively large populations (starting with around 10^3^–10^4^ individuals, and expanding to between 10^6^ and 10^7^ cells), stochasticity in the initial size is low, and we initialize all populations in the simulated ensemble with the same size ${n}_{0}={10}^{-3}$. Once the populations are formed, the choice of $s$ and $\alpha$ completely determines the population dynamics.

During the simulations, we record the average size $\bar{n}=\frac{1}{M}\sum _{i=1}^{M}{n}_{i}$ and the global producer fraction $\bar{x}$ across the metapopulation

5 $$\bar{x}=\frac{{\sum }_{i}{c}_{i}}{{\sum }_{i}({c}_{i}+{f}_{i})},$$

where ${n}_{i}$ and ${x}_{i}$ are the size and producer fraction of each population $i$, respectively. Note that this *global* fraction of public-good producers (i.e., the percentage of producer cells in the metapopulation) follows a different trajectory from the *local* one ${x}_{i}$ (the fraction of producers actually present in each of the component subpopulations). Specifically, while the latter always decreases – because producers grow more slowly than non-producers – the former can, in some cases, increase.

How $\bar{x}$ changes in time within a metapopulation, according to equations (4) (with $\alpha =200,s=0.05,\bar{n}={10}^{-3}$ is shown in Fig. 4a, and compositions sampled from the distribution in Fig. 4b, with average $\bar{x}(0)\simeq 0.33$); Supplementary Movie S1 shows the same data, together with the evolution of the joint distribution of sizes ${n}_{i}$ and compositions ${x}_{i}$. During early stages of growth, the more producers a population has, the quicker it accrues PVD, and the faster it grows. Populations with higher producer fractions rapidly increase their share in the metapopulation, driving up the global producer fraction $\bar{x}$. As time passes, populations with fewer producers also accumulate enough PVD to grow significantly (while the few with no producers never grow). Meanwhile, producer-rich populations have depleted their resources and end growth. As a result, the rate of increase of $\bar{x}$ first slows, then reaches a maximum ${\bar{x}}_{{\rm{\max }}}$ and decreases again. Finally, once all populations have entered the dormant state, the global producer fraction stabilizes. Its ultimate value depends on the production cost $s$ and, because all populations grow to the same size, it is lower or equal to the initial $\bar{x}(0)$.

The overall time course of $\bar{x}$ and $\bar{n}$ depends crucially on the choice of parameters, which reflect the features of the bacterial strains, as well as the cultivation conditions. Figure 4c, for example, shows how changing the growth reduction $s$ affects the magnitude of the increase in global producer fraction $\Delta \bar{x}={\bar{x}}_{{\rm{\max }}}-\bar{x}(0)$ (for $\alpha =200$ and the initial conditions shown in Fig. 4b). It is intuitively clear that a slower producer growth would yield a smaller increase. As the figure shows, we can find a region of extreme reduction ($s > 0.4$, which is unlikely to appear in natural systems), which cannot be offset by the benefit from the public good, thus producing no increase whatsoever in producer fraction. For lower values (roughly between 0.1 and 0.4), $\Delta \bar{x}$ is positive, and increases as $s$ is lowered. Finally, for low $s$ (below 0.1), $\Delta \bar{x}$ increases further, reaching values comparable with $\bar{x}(0)$, implying that the global producer fraction $\bar{x}$ almost doubles during growth, albeit transiently. The specific values of $s$ at which different results occur depend on the choice of $\alpha$ and of the ${x}_{{\rm{0}}}$ distribution. Nevertheless, the qualitative behavior of $\Delta \bar{x}$ remains the same.

The model thus provides insights into this public-good-mediated social interaction, and implies that it leads to a transient, but potentially very significant, increase in producer fraction. In the following section, we show that these predictions are in good agreement with experiments on competitive growth of mixed populations of producers and non-producers.

### Comparison between experimental and theoretical results

We grew mixed populations composed of producers KP1 and non-producers 3E2 under extreme iron limitation (KB/1 mM DP), in which PVD is indispensable for iron uptake and growth (see Supplementary Fig. S1). The metapopulation consisted of a 96-well plate (so the metapopulation size is $M$ = 96), and each well was inoculated with about 10^4^ cells. Producers and non-producers in each well were mixed in stochastic proportions, sampled from the distributions shown in Figs 4b and 5a, which were derived from the weighted average of three dice rolls (see Materials and Methods). These initial conditions mimic the characteristic variability of small populations. The mean initial producer fraction was $\bar{x}(0)\simeq 0.33$. As outlined in Fig. 1b, at given time points $t$, samples were taken from each well, merged, and their average cell number $\bar{N}(t)=\frac{1}{M}\sum _{i=1}^{M}{c}_{i}+{f}_{i}$ and mean global producer fraction $\bar{x}(t)$ were determined. Figure 5 shows the results of a representative experiment. On average, populations start growing after a lag phase of about 2 h and enter stationary phase after around 8 h. The global producer fraction $\bar{x}$ initially increases, up to a maximum ${\bar{x}}_{{\rm{\max }}}\simeq 0.5$. After sharply dipping to ${\bar{x}}_{{\rm{\min }}}\simeq 0.2$, it levels off to values around 0.2–0.3, and remains constant for at least 24 h. These results qualitatively agree with those obtained by solving equations (4) for an analogous metapopulation (see previous section and Fig. 4).

The only qualitative departure from the simulation results is that $\bar{x}$ drops towards the end of growth phase ($t\simeq 6\,$h) in the experiments. Notably, however, this also corresponds to an acceleration in population growth. Most probably, this stems from a change in the metabolic state of cells, which is not captured by the simplified description encoded in the equations (4).

We can also directly compare theoretical and experimental results. As initial conditions for the simulations, we sample the values of ${x}_{{\rm{0}}}$ from the same distribution as in the experiments, and set ${\bar{n}}_{0}={10}^{-3}$, which we estimated by dividing the mean minimum size from experiments (taken at the end of the lag phase, so as to eliminate the slight population decay) by the final yield. To set $s$, we considered that KP1 grows at a rate that is between 1.03 and 1.10 times lower than that for 3E2 (as determined previously), which corresponds to a range for $s$ of between 0.03 and 0.09. Since the rate of approach to saturation ${\alpha }$ reflects several complex processes, we opted to fit it.

**Figure 6:**
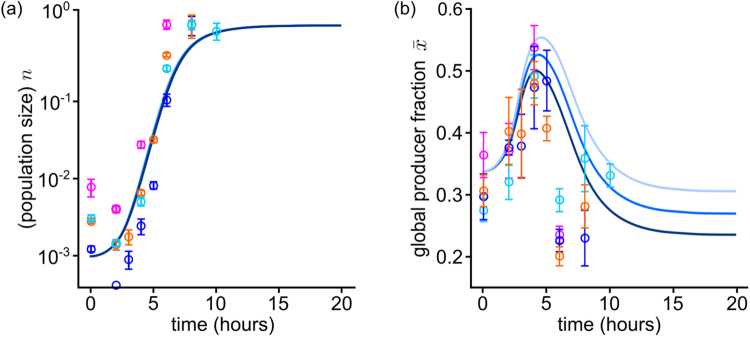
Comparison of simulation and experimental results for the population size (**a**) and global producer fraction (**b**) in a metapopulation. Solid lines represent numerical solutions of equations (4) for different values of the growth rate reduction $s$, and in (**b**) darker shades indicate higher values ($s\in \{0.03,0.05,0.07\}$). Dots of different colors indicate the results of different independent experimental runs. Error bars are the result of three to five determinations of the respective parameter at the given time point in one experiment. The population size $n$ is rescaled to the final yield (or carrying capacity). The stochastic initial compositions are sampled from the distribution in Fig. 4b. For appropriate values of the parameters (determined from fitting of the growth curve in (**a**)), theoretical and experimental result agree.

The data from four separate experiments (colored dots) and simulations for three possible values of $s$ and an appropriate saturation rate, ${\alpha }$ = 200 (solid lines) are shown in Fig. 6. To meaningfully compare the two sets of data, we also need to fix the global time scale of simulations, which is done by fitting the slope of the exponential phase in Fig. 6a. The increase in the global producer fraction observed in simulations agrees very well with experiment (Fig. 6b): $\bar{x}$ grows to a maximum ${\bar{x}}_{{\rm{\max }}}\simeq 0.5$ over similar periods, then decreases, and stabilizes to similar values.

Besides the aforementioned end-of-growth discrepancy – which seems to be due to behaviors well beyond the scope of our simplified mathematical description – experimental and theoretical results match.

## Discussion

In this work, we showed that social interactions mediated by a public good result in a transient increase in the global fraction of producers in a growing bacterial metapopulation. By combining theoretical modeling and experiments, we were able to quantitatively describe an exchange interaction involving a public good in a bacterial metapopulation.

We selected as our model system the native production of the iron-chelating siderophore pyoverdine (PVD) in *P. putida* KT2440 under iron limitation^41,47^. We characterized a constitutive producer (KP1) and a non-producer strain (3E2), and determined the growth rate reduction due to producing PVD. Under the chosen conditions, PVD is essential for iron acquisition and growth. We demonstrated that populations that produce more PVD grow faster than those with less (under otherwise identical conditions), though the magnitude of the benefit progressively diminishes as PVD accumulates, and eventually vanishes when the available iron ceases to limit growth. Based on these experimental facts, we constructed a set of differential equations that describes the growth of mixed populations of PVD producers and non-producers. Solving these equations for a large metapopulation, we found that, at first, the more producers (and thus more PVD) are present in the sub-populations, the faster they grow. This generates a positive covariance between composition and growth rate, which drives the global producer fraction up, in accordance with the Price equation^15,17,58^. As PVD accumulates, however, the benefit to cells eventually saturates, reducing the advantage enjoyed by these producer-rich populations; meanwhile, populations containing fewer producers begin to grow and ultimately catch up with the initially faster ones. Therefore, the increase in the global fraction of producers is transient, both in simulations and in experiments.

Previous experimental studies related similar phenomena to the so-called Simpson’s paradox^11,12^. However, they considered an artificial bacterial system, in which both the need for the public good and its production mechanism had been designed specifically for the experiments. In contrast, we employed a native system and quantified its social interactions, particularly the function and biochemical properties of the public good. Our analysis also shows that, without mechanisms to sustain it, the Simpson-related increase can only be transient. This conclusion is also compatible with previous qualitative predictions^13–16^, based on game theory models with implicit public goods. However, in contrast to our experiments, these studies predict that the producer fraction should peak at the end, instead of the mid-point of exponential growth. This indicates that simple cost-benefit considerations do not suffice to describe the social interaction. Inclusive fitness models have been used to describe an analogous scenario in wild-type *P. aeruginosa*, reaching qualitative conclusions compatible with our results^29,30^. Similarly to game-theoretical approaches, however, they remain mainly conceptual^59^. Our systems biology approach, instead, provides a simple description, with testable quantitative predictions, as well as important insights into the social interaction.

In metapopulation settings, diffusion, dispersal, and mobility affect public good interactions^30,60^. Besides these factors, our results highlight the potential role of the timing of dispersal. Some studies already pointed to dispersal timing, by considering a metapopulation that periodically splits into groups and merging these again to reform the pool. After several cycles, the metapopulation might develop stable coexistence of the strains^13–16^, or even have the producers fixate^11,12^. Testing this process, however, requires Poisson dilution conditions which implicate very low initial densities of producer cells. As a consequence, large fractions of cells die under iron-limiting conditions before physiologically effective PVD concentrations are reached. Therefore, a repetitive scenario of group formation and merging is experimentally not feasible for our well mixed cultivation conditions. In principle, introduction of a non-selective growth phase may rescue such a scenario^61^.

An interesting next step will be to include regulatory aspects in our system. Like many other bacteria, the wild-type *P. putida* KT2440 continually senses changes in environmental conditions, and uses this information to tune production of the public good^62–64^. By employing constitutive producer strains, we shifted the focus more on the social role of PVD itself, while replicating a potential earlier stage of evolution (if PVD production evolved before regulation). Our model also indicates that a cost-saving strategy such as down-regulation of PVD production as a consequence of PVD accumulation is not sufficient to prevent the long-term decline of the global fraction of producers, because all populations with producers eventually accumulate the same PVD concentration. So accounting for regulation, which has been shown to also impact growth^65^, will also necessarily involve elaborate production curves^63^ and cost-saving strategies^66^. Ultimately, adaptive production raises complex questions about how cells shape the ecological and environmental conditions in which they interact^67^.

Another possible extension would be to allow privatized use of the public good. Privatized use of siderophores, in particular, has been shown to introduce fascinating social dynamics into intra- and inter-species competition^35,68–70^. Limited diffusion and private use have important social consequences^32,33^. Indeed, several studies have intensely debated under what conditions the secreted siderophores actually behave as public goods^42,71–73^. In our conditions, however, populations seem to behave as well-mixed, with negligible privatization.

Taken together, our work uses a simplified setting to highlight the determinant role of public goods in social interactions and population dynamics. For example, we showed the profound consequences of the public good’s accumulation and saturating benefits, which simple game-theoretical considerations would fail to describe. Our approach could clearly be extended to investigate the fundamental principles underlying different interactions and bacterial systems. Thereby it should stimulate more mechanistic analyses of bacterial social interactions and their impact on population development.

## Materials and Methods

### Strains and growth conditions

*Escherichia coli* DH*5α* [F- φ80d *lacZ* ΔM15 Δ(*lacZYA-argF*) U169 *deoR recA1 endA1 hsd* R17(rk−, mk+) *phoA supE44* λ- *thi-1 gyrA96 relA1*] was used as the carrier for plasmids. *Pseudomonas putida* KT2440 and the derived strain 3E2 (non-producer)^47^ were employed as PVD producer (wild-type) and non-producer, respectively. *E. coli* strains were grown in lysogeny broth (LB) at 37 °C, and *P. putida* strains were grown at 30 °C in King’s medium B (KB)^74^. KB medium was supplemented with 100 µM FeCl_3_ and/or 1 mM of the iron chelator 2,2′-dipyridyl (DP) where indicated. Solid media were LB or KB with 1.5% agar.

### Generation of the constitutive PVD producer strain KP1

A *P. putida* strain that constitutively produces PVD was generated by placing a copy of the *pfrI* gene under the control of the constitutive promoter P_*A1/04/03*_^54^. For this purpose, P_*A1/04/03*_ and the *pfrI* gene were amplified by PCR from the plasmid mini*Tn*7(Gm)P_A1/04/03_ecfp-a^75^ and the *P. putida* genome, respectively, cloned into plasmid pUC18R6K-mini-*Tn*7T-Gm, and inserted at the *att*Tn7 site in *P. putida* KT2440 following a mini-*Tn*7 protocol for *Pseudomonas*^76^. The resulting *P. putida* strain KP1 was verified by PCR amplification of corresponding genome regions and sequencing. All oligonucleotide primers used for strain generations and verification are listed in Supplementary Table S2.

### Quantitative analysis of PVD production

Pre-cultures of the respective strains were grown in iron-replete medium (KB/200 µM FeCl_3_) at 30 °C for 18 h. The pre-cultures were used to inoculate the appropriate media for the growth of the cultures used in experiments (${N}_{0}={10}^{7}$ cells mL^−1^). Experiments were performed in 24-well plates (2 mL culture/well). The plates were shaken at 300 rpm at 30 °C. At given time points samples were taken and the optical density at 600 nm was measured. Subsequently, cells were removed by centrifugation, and the relative PVD content was determined by measuring the fluorescence emission at 460 nm (excitation 400 nm). PVD production was analyzed under iron limitation (KB/1 mM DP; KB/100 µM FeCl_3_/1 mM DP) and iron replete conditions (KB; KB/100 µM FeCl_3_). Each individual experiment was performed with three parallel replicates. A minimum of three independent experiments were conducted per condition.

### Growth characteristics of strains under different environmental conditions

Pre-cultures of the respective strains were grown in iron-replete medium as described above for the analysis of PVD production, and used to inoculate the appropriate media for growth of the cultures used in experiments (${N}_{0}={10}^{7}$ cells mL^−1^). Experiments were performed in 96-well plates (150 µL culture/well). The plates were shaken at 300 rpm at 30 °C. Growth was followed by measuring the optical density at 600 nm using a microplate reader (Infinite^®^ M200 from Tecan Trading AG). The measurement was controlled and monitored with the i-control™ Software from Tecan Trading AG (30 °C, shaking at 280 rpm, 880 s per cycle, minimum 80 cycles). Each condition was implemented in six replicates per experiment, including medium blanks. For low cell numbers (e.g., ${N}_{0}={10}^{4}$ cells 150 µL^−1^), growth was analyzed by determining colony forming units (*cfu*) over time (threefold per time point). The specific growth rate $\mu$ represents a quantitative measure of growth in the exponential phase and was calculated using the following equation: $\mu =\frac{\mathrm{ln}({N}_{t2}-{N}_{t1})}{t2-t1}$.

### Quantitative assessment of the benefit of PVD

The benefit conferred by PVD was quantified under iron-limiting conditions (KB/1 mM DP) with the non-producer strain 3E2. PVD was isolated according to a previously described protocol^77^ and added to the medium at concentrations of between 0 and 20 µM. Growth was monitored via optical density measurement, and the specific growth rate $\mu$ was calculated as described in the previous paragraph. Each individual experiment was performed with four parallel repeats per PVD concentration, and three independent experiments of this type were conducted per PVD concentration.

### Determination of PVD sharing in mixed culture

Cells were grown in KB/1 mM DP (initial producer frequency $\bar{x}(0)\simeq 0.33$, ${N}_{0}={10}^{4}$ cells/150 µL, 96-well plate format) at 30 °C. Colony forming units (*cfu*) were determined at given time points (five replicates per time point), and producer and non-producer cells were discriminated by colony color and size. Three independent experiments were performed, each yielding similar results.

### Stability of PVD in KB medium with and without bacteria

Medium without cells and medium containing about 10^7^ cells mL^−1^ of the non-producer were supplemented with 2 µM PVD and incubated at 30 °C for 48 h. At given time points samples were taken, and the relative PVD contents of medium and of the supernatant of medium with cells were determined by measuring the fluorescence emission of PVD at 460 nm (excitation 400 nm).

### Competitive growth experiments

To analyze the impact of the initial producer frequencies $${x}_{{\rm{0}}}$$ on growth, strains KP1 and 3E2 were mixed in KB/1 mM DP (96-well plate, $${N}_{0}={10}^{4}$$ cells 150 µL^−1^, $${x}_{0}\,\in \,\{0,\,0.1,$$$$0.2,\,0.3,\,0.5,\,0.75,\,1.0\}$$). Total cell numbers were determined at the end of the lag phase and after 8 h of shaking at 30 °C by counting *cfu*. For each condition, a minimum of three individual experiments were performed. To analyze the development of the total cell number $$\bar{N}(t)$$ and global producer frequency $$\bar{x}(t)$$ in metapopulations, a random distribution of the initial producer frequency $${x}_{0}$$ was established by rolling three dices. The values of each triplet were weighted (lowest 2/3, middle 2/9 and highest 1/9) and rounded to yield sixteen different values from 0 to 15 that are equivalent to sixteen different initial producer frequencies $${x}_{{\rm{0}}}$$ ranging from 0 to 1.0 and result in an initial average global cooperator fraction $$\bar{x}(0)$$ of about 0.33. Cells were grown in KB/1 mM DP (96-well plate, $${N}_{0}={10}^{4}$$ cells 150 µL^−1^) at 30 °C while shaking at 300 rpm. At given time points aliquots of each well were merged and $$\bar{N}(t)$$ was determined by counting *cfu*. The global producer frequency $$\bar{x}(t)$$ was obtained based on differences in the color and size of the colonies of KP1 and 3E2 on KB agar plates (minimum three replicates per time point).

### Stochastic initial conditions and ensemble averages

We generate all triplets of the integers between 0 and 5 to simulate the results of a sequence of (simultaneous) throws of three dice. Since the weights in the experimental procedure are assigned based on the order of the rolled values, we order the “rolled” values within each triplet from lowest to highest. This results in a table of all possible 3-dice rolls, which we can use directly to generate the initial conditions and simulate equations (4). To speed up calculations, however, we remove duplicate triplets: for example, 113, 131, 311 are different triplets before sorting, but are the same after. Once we remove the duplicate combinations, we assign the appropriate probability to them, i.e. the number of ways to produce them before sorting divided by the total number of triplets. With a minimal combinatorics, one can compute the total number of triplets (${6}^{3}=216$), and the multiplicities of triplets: those with three equal values have only one way to appear before sorting; those with two equal values have three; those with all different values have six.

To simulate the time evolution of $n$ and $x$, we generate the initial composition ${x}_{{\rm{0}}}$ for each triplet, using the weighted average described above. After setting ${n}_{{\rm{0}}}$, ${\alpha }$, and $s$, the temporal evolution of the average $\bar{x}$ and $\bar{n}$ in ensembles of populations can be computed by solving equations (4) for each of the allowed values of ${x}_{{\rm{0}}}$ and weighting it using the relative probability, computed as described above.

### Data Availability

The data that support the findings of this study are available from the corresponding authors upon reasonable request.

## Electronic supplementary material

Supplementary Information

Movie S1
