# Supplementary material for: Interactions mediated by a public good transiently increase cooperativity in growing Pseudomonas putida metapopulations
Source: Sci Rep. 2018 Mar 6;8:4093. doi: 10.1038/s41598-018-22306-9 (PMC5840296; doi:10.1038/s41598-018-22306-9)
Supplement: Supplementary file 1 — Supplementary Information [file 41598_2018_22306_MOESM1_ESM.docx]

**Interactions mediated by a public good transiently increase cooperativity in growing *Pseudomonas putida* metapopulations**

Felix Becker,^a^ Karl Wienand,^b^ Matthias Lechner,^b^ Erwin Frey,^b,*^ Heinrich Jung^a,*^

^a^Microbiology, Department Biology 1, Ludwig-Maximilians-Universität Munich, Grosshaderner Strasse 2-4, Martinsried, Germany; ^b^Arnold-Sommerfeld-Center for Theoretical Physics and Center for Nanoscience, Ludwig-Maximilians-Universität, Theresienstrasse 37, D-80333 Munich, Germany

^*^Correspondence to: Heinrich Jung, [hjung@lmu.de](mailto:hjung@lmu.de) (HJ) and Erwin Frey [frey@lmu.de](mailto:frey@lmu.de) (EF).

FB and KW contributed equally to this work.

# Supplementary Information

# Notes

# Derivation of the growth rate $\boldsymbol{\mu(p)}$

Assume a cell is born with an internal iron concentration ${Fe}_{\text{in}}(0)$ and a volume $V(0)$. Let $p$ be the concentration of PVD-Fe complexes (which we take to be the same as that of PVD; see text). Each cell, then, incorporates iron ions at a constant rate $kp$ that is proportional to the concentration of PVD. So at time $t$ after its birth, the cell has accumulated $kpt$ iron ions. Its internal iron concentration ${Fe}_{\text{in}}(t)$, then, is ${Fe}_{\text{in}}(0)V(0)$ (that is, the number of iron atoms at birth), plus the iron it has collected, all divided by the volume birth $V(t)$ it has reached:

${Fe}_{\text{in}}(t)= \frac{{Fe}_{\text{in}}(0)V(0)+kpt}{V(t)}$ .

Because cells try to maintain iron concentration homeostasis, we can consider ${Fe}_{\text{in}}$ to be constant. Moreover, as long as iron is the limiting factor for growth, the growth rate $\mu(p)$ depends only on the PVD concentration.

On average, cells divide at time $t_{D}=1/\mu(p)$, given that growth is logistic. Moreover, at the moment of division, the cell has attained twice the volume of its future daughters: $V(t_{D})=2V(0)$. With these substitutions in the above equation, and minimal algebra, we obtain equation (2).

# Estimation of iron incorporated into cells

The iron content of a bacterial cell ranges from $\sim{10}^{5}$ to ${10}^{6}$ atoms per cell^1,2^. In our experimental setup, cells reach a maximum density of ${2\times10}^{7}$ cells per 150 µL at the end of the exponential growth phase accumulating in total ${2\times10}^{12}$ to ${2\times10}^{13}$ iron atoms. We determined the iron concentration of our KB preparation by atomic absorption spectroscopy and found a concentration of ~8 µM (corresponding to $\sim{7.2\times10}^{14}$ iron atoms per 150 µL KB medium). Using these numbers we calculated that ~0.28 to 2.8% of the total iron of KB is incorporated into cells by the end of the exponential growth phase.

**References**

1 Abdul-Tehrani, H. *et al.* Ferritin mutants of *Escherichia coli* are iron deficient and growth impaired, and *fur* mutants are iron deficient. *J Bacteriol* **181**, 1415-1428 (1999).

2 Andrews, S. C., Robinson, A. K. & Rodriguez-Quinones, F. Bacterial iron homeostasis. *FEMS Microbiol Rev* **27**, 215-237 (2003).

**
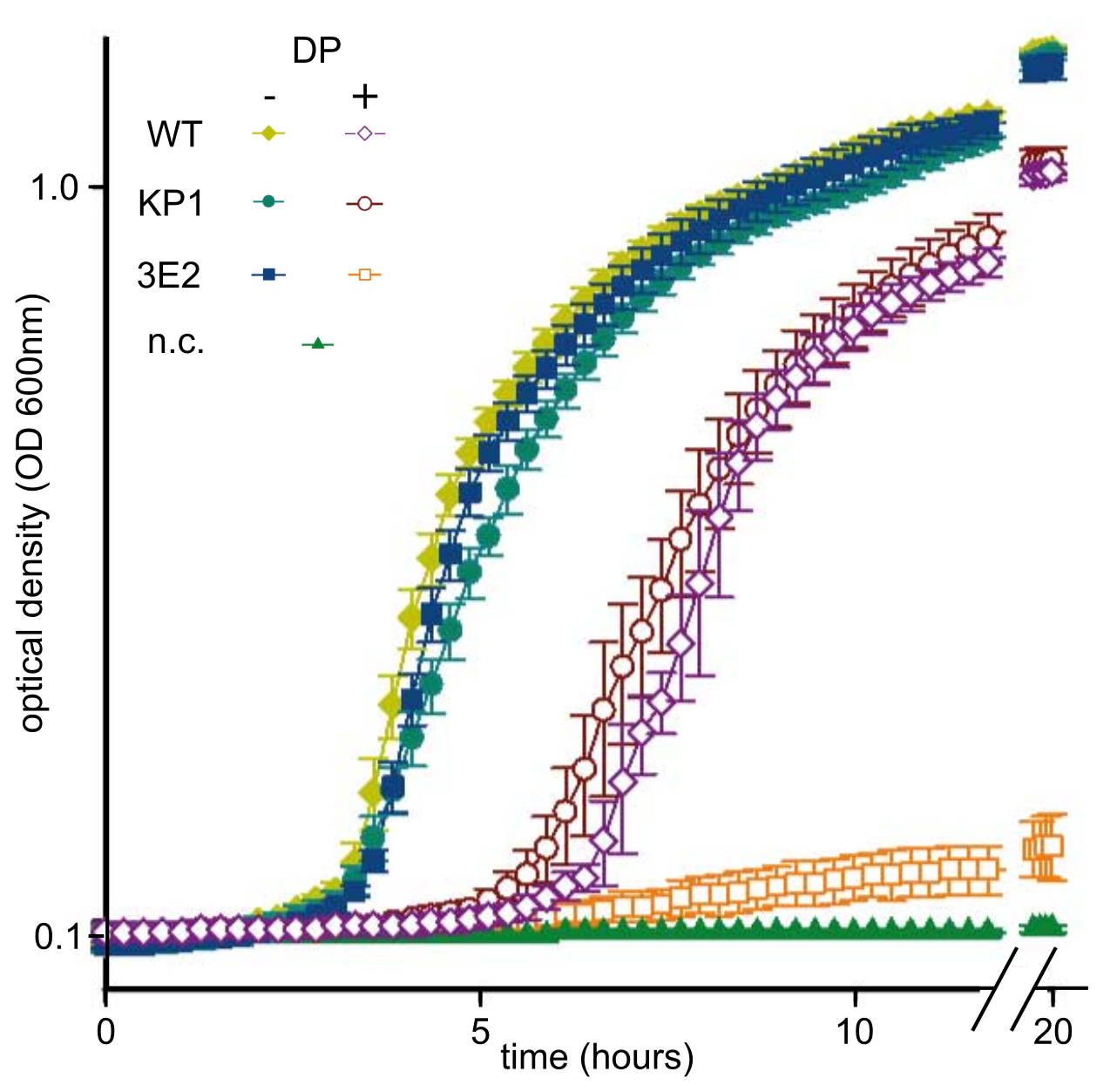
**

**Figure S1.** Growth of *P. putida* KT2440 and the derived strains KP1 and 3E2 under iron replete and limiting conditions. Cells were grown in KB supplemented with iron (KB/100 µM FeCl_3_, full symbols) and in KB with iron chelator DP (KB/100 µM FeCl_3_/1 mM DP, empty symbols). When iron is more available, PVD is not needed for growth, 3E2 and WT grow about at the same rate, and KP1 grows slower. Under iron limitation, producing strains (KP1 and WT) benefit from production and grow much faster than non-producing 3E2. The experiment was performed as described in the legend of Fig. 3a.

**
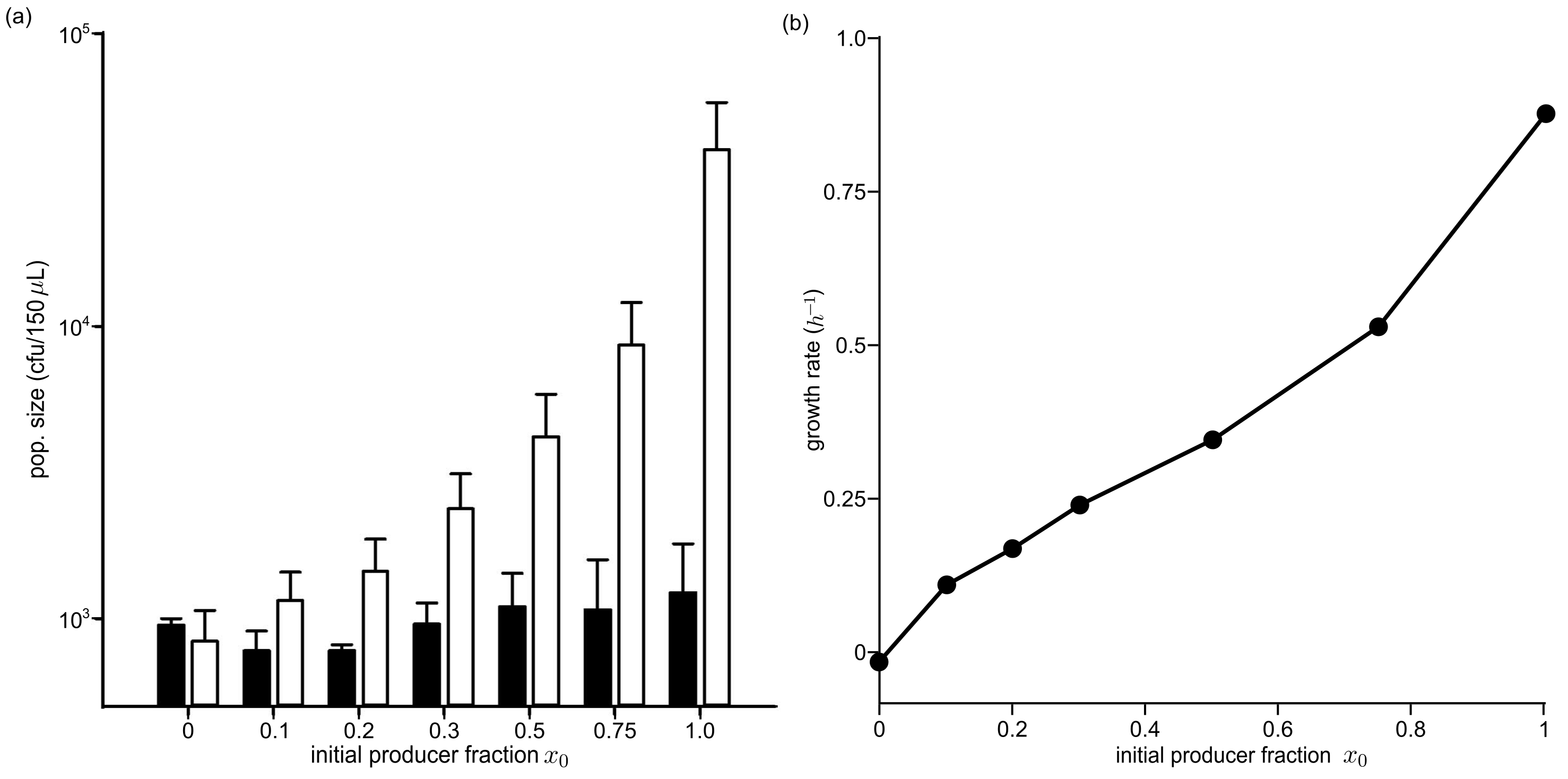
**

**Figure S2.** Impact of the initial producer fraction *x_0_* on the growth of mixed populations under iron limiting conditions. **(a)** Impact of the initial producer fraction *x_0_* on the growth yield. Strains KP1 and 3E2 were grown in mixed culture under iron-limiting conditions (KB/1 mM DP, $N_{0}$ about ${10}^{3}$ cells/150 µL, 96-well plate format) with the given initial producer frequencies $x_{0}$. Total cell numbers were determined by counting *cfu* at the beginning of the experiment (black columns) and after 8 h of incubation (weight columns). For each condition, minimum three individual experiments were performed. (**b**) Impact of the initial producer fraction $x_{0}$ on the specific growth rate $\mu$. Mixed cultures of strains KP1 and 3E2 with $x_{0}$ values between 0 (=100% 3E2) and 1 (=100% KP1) were incubated in shaking 96-well microtiter plates at 30^o^C ($N_{0}={10}^{7}$ cells mL^-1^). Growth was analyzed by measuring the optical density at 600 nm using a Tecan microplate reader. $\mu$ was determined for each condition from the exponential phase of the resulting growth curves. All growth parameters represent the means of five growth experiments. Deviations were <10% of the mean value.


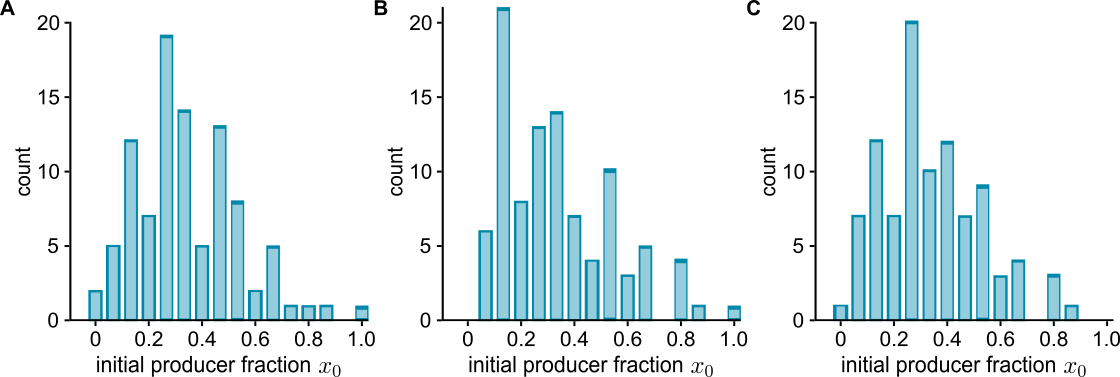


**Figure S3.** Distribution of initial compositions $x_{0}$ in three replicates of the experiment. Although specific values differ, the overall features of the distribution remains the same. Most populations start mixed, with $x_{0}$ between 0.1 and 0.5. When at all present, populations with all producers or no producers are very rare.

# Table S1. Specific growth rate of *P. putida* KT2440 (WT), the non-producer (3E2), and the constitutive PVD producer (KP1) under iron-rich and iron-limiting conditions.

| **Growth medium** | ***µ_WT_***  ***(h^-1^)*** | ***µ_3E2_***  ***(h^-1^)*** | ***µ_KP1_***  ***(h^-1^)*** | ***µ_3E2_/µ_KP1_*** |
| --- | --- | --- | --- | --- |
| KB^a^ | 0.797 ± 0.047 | 0.786 ± 0.034 | 0.732 ± 0.22 | 1.074 |
| KB/100 µM FeCl_3_^a^ | 0.766 ± 0.013 | 0.759 ± 0.054 | 0.701 ± 0.027 | 1.082 |
| KB/DP^a^ | 0.389 ± 0.178 | 0.004 ± 0.001 | 0.576 ± 0.011 | 0.007 |
| KB/100 µM FeCl_3_/DP^a^ | 0.615 ± 0.055 | 0.025 ± 0.007 | 0.582 ± 0.061 | 0.043 |
| KB/100 µM FeCl_3_^b^ | 1.237 ± 0.056 | 1.238 ± 0.065 | 1.201 ± 0.064 | 1.031 |
| KB/100 µM FeCl_3_^c^ | n.d. | 1.182 ± 0.099 | 1.077 ± 0.060 | 1.097 |

^a^The specific growth rate $\mu$ was calculated from the growth curves shown in Fig. 3a and Fig. S1. Cells were grown in shaking 96-well microtiter plates at 30^o^C ($N_{0}={10}^{7}$cells mL^-1^). Growth was analyzed by measuring the optical density at 600 nm using a Tecan microplate reader. Mean and SD values were calculated from fifteen growth experiments. The large error of *µ_WT_* tin KB/DP was attributed to cell aggregation interfering with a reliable detection of optical densities (cp. also Fig. 3a).

^b^Cells were grown in shaking 24-well microtiter plates at 30^o^C ($N_{0}={10}^{7}$cells mL^-1^). Every hour 50% of the culture was replaced with fresh medium. Growth was analyzed by measuring the optical density at 600 nm using a 1-mL cuvette (d=1 cm). Mean and SD values were calculated from nine growth experiments.

^c^Cells were grown in shaking 96-well microtiter plates at 30^o^C ($N_{0}={10}^{4}$cells mL^-1^). Growth was analyzed by determination of colony forming units, *cfu*. Mean and SD values were calculated from five growth experiments.

# Table S2. Oligonucleotides used in this investigation

| **Name** | **Sequence (5'...3')** |
| --- | --- |
|  |  |
| **Generation of *P. putida* KP1** |  |
| P*_A1_04_03_* bw kpn | AAATAGGGGGGTACCCGCACATTTCCC |
| P*_A1_04_03_* mod2 | TTCCGCCATGCTTAATTTCTCCTCTTT |
| *pfrI* start mod2 | AAATTAAGCATGGCGGAACAACTATCC |
| *pfrI* end mod2 | TGCGGCGTTGGATCCGCTGCGAGTTATTGGCCG |
|  |  |
| **Sequencing insert on plasmid** |  |
| mini Tn7 reverse MCS | TTGCATTACAGTTTACGAACCGAAC |
|  |  |
| **Sequencing Tn7 insertion on genome** |  |
| checkdown primer trans | GTCTTATTACGTGGCCGTGC |
| Primer TN7R as | CCACGCCCCTCTTTAATACG |
| tn7left s | TTTGTCATTTTTAATTTTCG |
| checkup primer trans | GCAGGAGCCGATGAGACAGA |

**Movie S1.** Temporal evolution of $\bar{x}$in a metapopulation. The evolution of $\bar{x}$was obtained by solving equations (4) together with the evolution of the joint distribution of sizes $n_{i}$ and compositions $x_{i}$.
